# Study on the effects of the different polar group of EPA-enriched phospholipids on the proliferation and apoptosis in 95D cells

**DOI:** 10.1007/s42995-021-00097-9

**Published:** 2021-06-07

**Authors:** Yao Guo, Qin Zhao, Yingying Tian, Yuanyuan Liu, Ziyi Yan, Changhu Xue, Jingfeng Wang

**Affiliations:** 1 College of Food Science and Engineering, Ocean University of China, Qingdao, 266003 China; 2 School of Food Engineering, Ludong University, Yantai, 264025 China; 3 Marine Biomedical Research Institute of Qingdao, Qingdao, 266061 China; 4 Laboratory for Marine Drugs and Bioproducts, Pilot National Laboratory for Marine Science and Technology (Qingdao), Qingdao, 266237 China

**Keywords:** Marine phospholipid, EPA-PC, EPA-PE, Proliferation, Apoptosis, Lung cancer

## Abstract

EPA-enriched phosphatidylcholine (EPA-PC) and EPA-enriched phosphatidylethanolamine (EPA-PE) are newly identified marine phospholipids. The polar group of phospholipids is known to influence EPA-phospholipid activity. However, the differences in anti-tumor effects between EPA-PC and EPA-PE have not been reported. In this study, we evaluated the effects of two forms of EPA on the proliferation and apoptosis in the lung-cancer cell line 95D as well as possible molecular mechanisms. Our results showed that EPA-PC effectively inhibited proliferative activity and promoted apoptosis of 95D cells in a dose-dependent manner, while EPA-PE had no effect on cell proliferation, although it slightly promoted apoptosis. Western blot results showed that EPA-PC and EPA-PE upregulated the expression of PPARγ, RXRα, and PTEN, and downregulated the PI3K/AKT signaling pathway. Furthermore, EPA-PC and EPA-PE induced the expression of the pro-apoptotic gene, Bax, and reduced the expression of the anti-apoptotic gene, Bcl-xl. Additionally, EPA-PC and EPA-PE promoted the release of cytochrome c and activated the apoptotic enzyme-cleaved caspase-3. These data suggest that the anti-tumor effect of EPA-phospholipids may be exerted via a PPARγ-related mechanism. EPA-PC was more efficacious as compared to EPA-PE, which might be due to the different polar groups of phospholipids.

## Introduction

Cancer is the leading causing of death in over 91 countries. Worldwide, in 2018, approximately 18.1 million people were newly diagnosed and 9.6 million died (Nasim et al. 2018). Although scientists have made advancements in cancer treatment strategies, many cancers are still insurmountable and present with poor prognosis and high mortality (Petpiroon et al. 2019). More importantly, reduced sensitivity to radiotherapy and chemotherapy and a high probability of recurrence and metastasis are also critical issues (Shen et al. 2019). Nutritional intervention adjuvant therapy is gaining popularity in cancer treatment. Abdi et al. (2014) found that omega-3 fatty acids could enhance the sensitivity of multiple myeloma cells to the anti-tumor drug, bortezomib.

The peroxisome-proliferation-activated receptor gamma (PPARγ) is a member of the nuclear receptor family and a key transcriptional regulator of fatty acid and glucose metabolism (Sascha 2015). Recently, substantial evidence indicates that PPARγ has the characteristics of a tumor suppressor. PPARγ is expressed in many cancers including those of the colon, breast, and prostate, and PPARγ ligands are generally found to have anti-proliferative effects in these cells (Lehrke et al. 2005). The loss of PPARγ may create a pro-tumorigenic microenvironment (Apostoli et al. 2013). Studies have shown that PPARγ agonists can inhibit the growth of a variety of cancer cells in vitro, and in combination with other chemotherapeutic drugs, can enhance the anti-tumor effect of the drugs. This effect could be reversed by the PPARγ inhibitor, GW9662 (Jiang et al. 2015; Skelhorne-Gross et al. 2012).

**Fig. 1:**
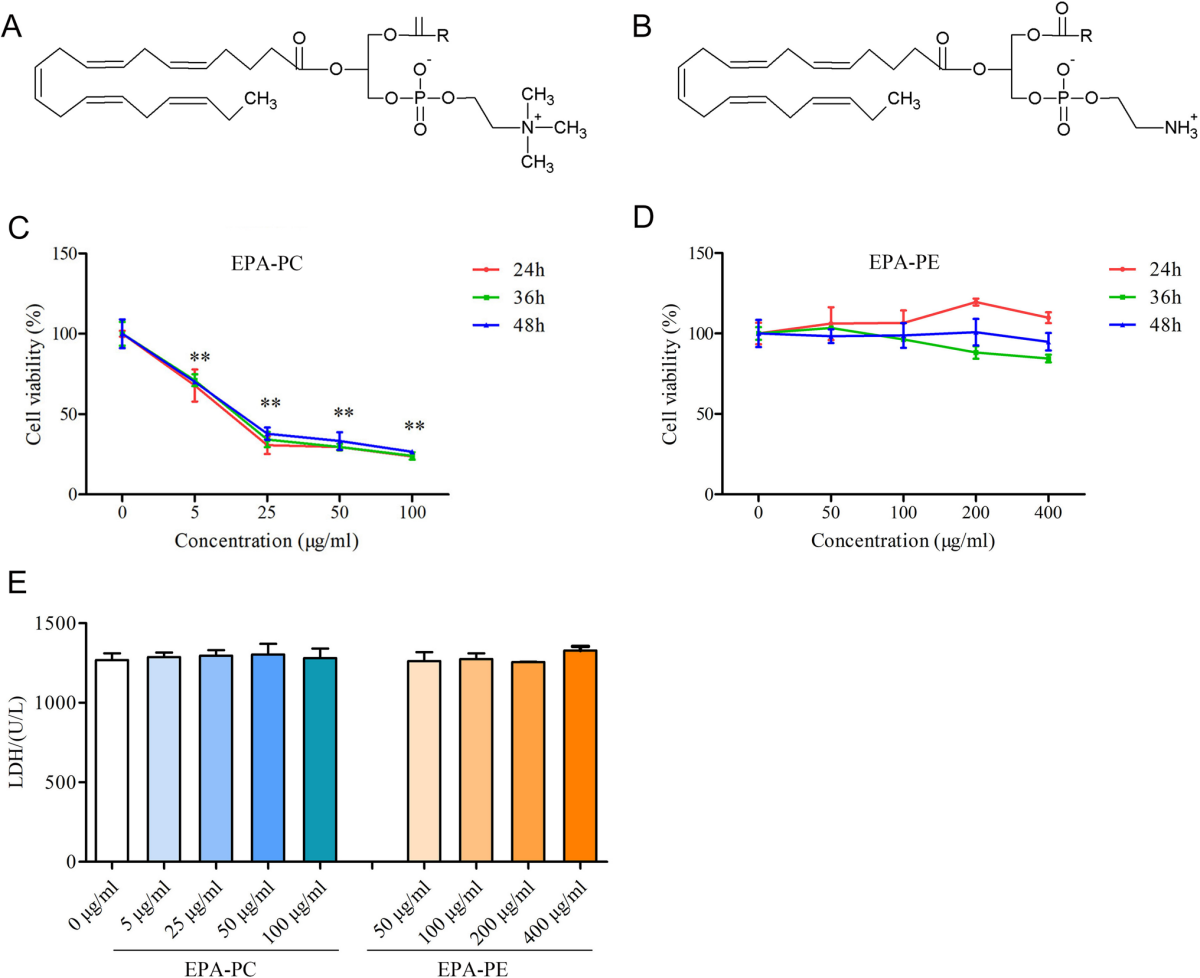
Effect of EPA-PC and EPA-PE on the growth of 95D lung-cancer cells. **a** The general structure of EPA-PC. **b** The general structure of EPA-PE. **c** 95D cells were treated with different concentrations of EPA-PC for 24, 36, and 48 h. **d** 95D cells were treated with different concentrations of EPA-PE for 24, 36, and 48 h. Cell viability was determined by MTT assay. **e** Effects of EPA-PC and EPA-PE on LDH activity in supernatant. **P < 0.01 versus control group. The data are presented as the mean ± SD of three separate experiments

Eicosapentaenoic acid (EPA) is an omega-3 long-chained polyunsaturated fatty acid that exerts many physiological effects (Acharya et al. 2019; Bonnet and Ferrari 2011; Peng et al. 2020; Soni et al. 2019). For example, a large number of studies have reported its anti-tumor effect. Study authors reported that EPA could inhibit proliferation, promote apoptosis and autophagy of cancer cells (Zhang et al. 2007; Zheng et al. 2017), and effectively inhibit the growth and metastasis of tumors (Eltweri et al. 2016). In recent years, EPA has been proven to be a natural ligand for PPARγ (Lehrke et al. 2005), which may explain its anti-tumor effect. EPA-enriched phospholipids (PLs) are newly discovered marine phospholipids that contain abundant EPA in the sn-2 position of the carbon skeleton. Examples of this class of compounds include EPA-enriched phosphatidylcholine (EPA-PC), EPA-enriched phosphatidylethanolamine (EPA-PE), and EPA-enriched phosphatidylserine (EPA-PS). The general structures of EPA-PC and EPA-PE are shown in Fig. 1a, b. The different polar groups at the sn-3 position of the phospholipid glyceryl group are responsible for differences in nutritional properties among these compounds. Zhou et al. (2018a, b) reported that EPA-PS shows better improvement effects than EPA-PC/PE on neurotrophic activity. They also point out that these effects might be attributed to the polar phospholipid groups. Data on the bioavailability and biological effects of different forms of EPA can help people choose more nutritional foods to obtain necessary fatty acids. However, there are no studies comparing the anti-tumor activity of EPA-PC and EPA-PE.

In the present study, we compared the effects of different forms of EPA on the proliferation and apoptosis of 95D lung-cancer cells. We also studied their molecular mechanisms in vitro to provide theoretical support for nutritional adjuvant therapy for cancer and for the high-value utilization of sea cucumbers.

## Results

### Effect of EPA-PC and EPA-PE on the proliferation of 95D cells

We first examined the effects of EPA-PC and EPA-PE on the proliferation of 95D cells. As shown in Fig. 1c, EPA-PC significantly inhibited the growth of 95D cells in a dose-dependent manner. The IC_50_ value of EPA-PC for 95D cells at 48 h was determined to be 15.97 μg/ml. However, EPA-PE had no significant effect on the proliferation of 95D cells (Fig. 1d). In addition, neither EPA-PC nor EPA-PE affected LDH activity in the cell supernatant (Fig. 1e), indicating that they were not cytotoxic.

### Effect of EPA-PC and EPA-PE on the morphology of 95D cells

**Fig. 2:**
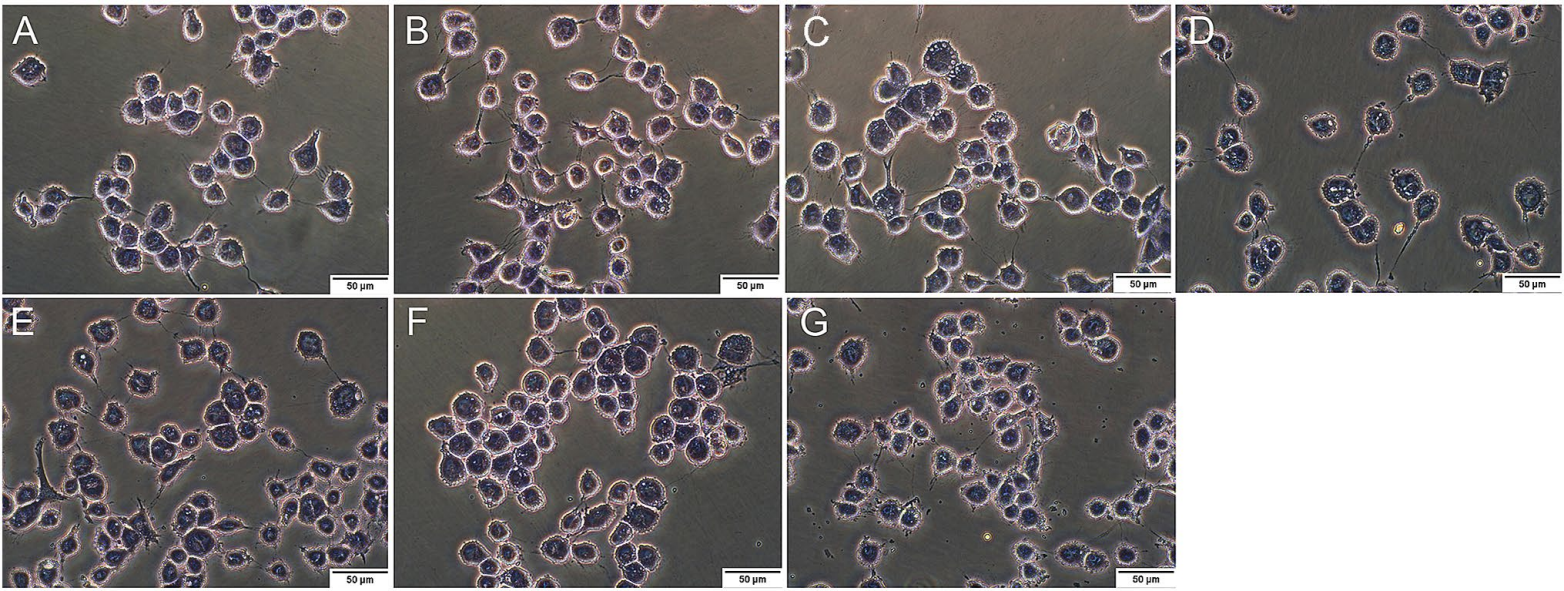
Effect of EPA-PC and EPA-PE on morphology of 95D cells. 95D cells were treated with different concentrations of EPA-PC and EPA-PE for 24 h. **a** 0 μg/ml; **b** EPA-PC: 25 μg/ml; **c** EPA-PC: 50 μg/ml; **d** EPA-PC: 100 μg/ml; **e** EPA-PE: 25 μg/ml; **f** EPA-PE: 50 μg/ml; **g** EPA-PE: 100 μg/ml. The morphology of the cells was observed under microscope (magnification 400 ×) after hematoxylin staining

To investigate the effect of EPA-PC and EPA-PE on the morphology of lung-cancer cells, morphological changes of the cells were observed using hematoxylin staining. As shown in Fig. 2, control group cells displayed normal morphology and adherence. Compared with the control group, many vacuoles appeared in the cells after the EPA-PC intervention and their number increased with increasing doses. After the EPA-PE intervention, a few vacuoles appeared in 95D cells. These results suggested that EPA-PC and EPA-PE could induce morphological changes and destroy the cell membranes of 95D cells.

### Effect of EPA-PC and EPA-PE on the apoptosis of 95D cells

**Fig. 3:**
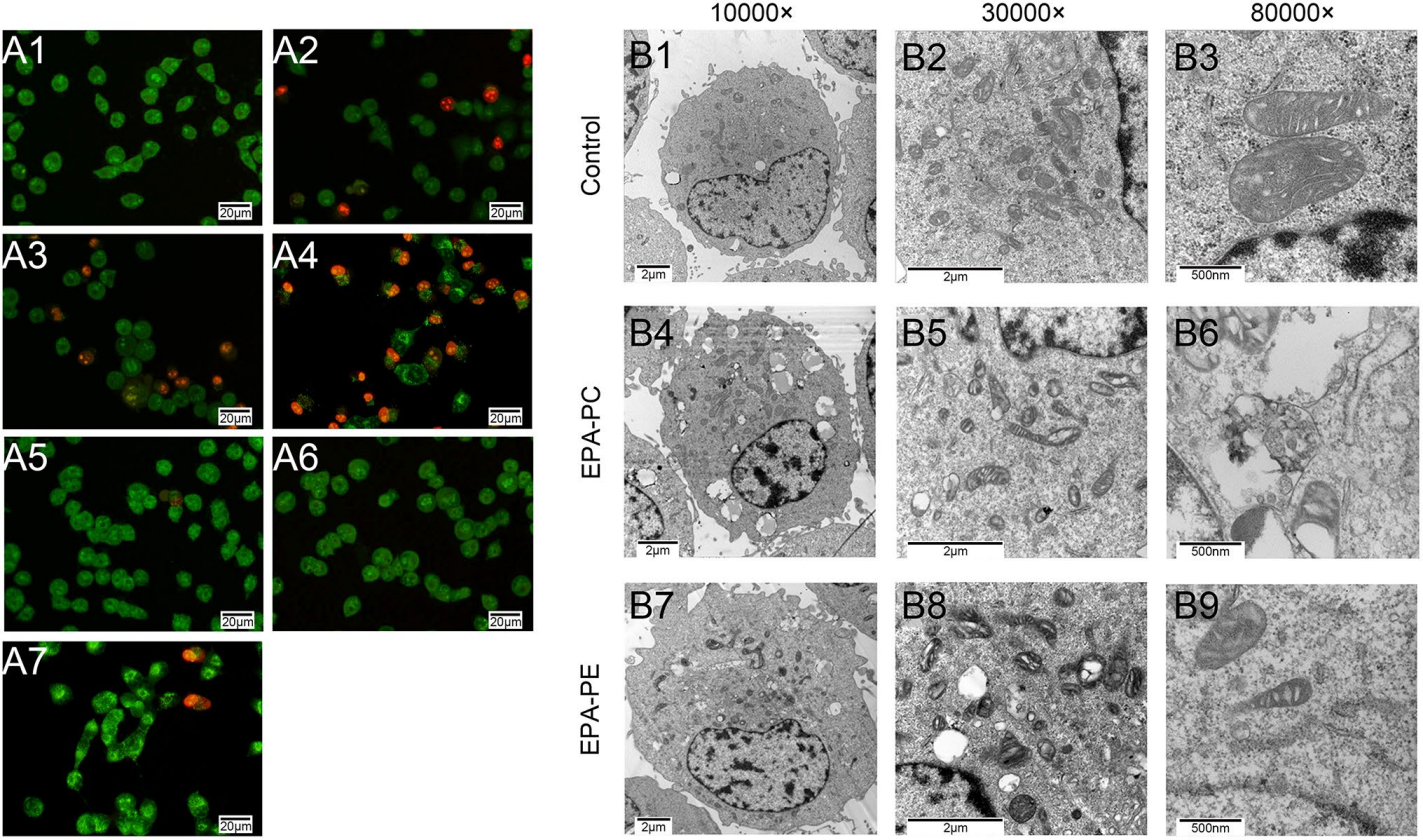
Effect of EPA-PC and EPA-PE on apoptosis of 95D cells. **a** AO/EB staining of 95Dcells after treated with different concentrations of EPA-PC or EPA-PE for 24 h (magnification 400 ×). A1: 0 μg/ml; A2: EPA-PC, 25 μg/ml; A3: EPA-PC, 50 μg/ml; A4: EPA-PC, 100 μg/ml; A5: EPA-PE, 25 μg/ml; A6: EPA-PE, 50 μg/ml; A7: EPA-PE, 100 μg/ml. **b** TEM was used to further study the apoptotic morphology of 95D cells (Column 1: magnification 10000 ×. Column 2: magnification 30000 ×. Column 3: magnification 80000 ×)

Cell apoptosis plays an important role in restraining tumor progression. To investigate the effect of EPA-PC and EPA-PE on the apoptosis of 95D cells, acridine orange and ethidium bromide (AO/EB) staining was carried out. Fluorescence microscopy revealed that control cells appeared uniformly green, early apoptotic cells appeared pale orange, and late apoptotic cells appeared red. As shown in Fig. 3a, EPA-PC intervention significantly increased the number of early and late apoptosis cells, while EPA-PE only slightly promoted the apoptosis process of 95D cells at higher concentrations.

To further study morphological evidence of apoptosis in treated cells, TEM was performed to observe the submicroscopic structure of cells. As shown in Fig. 3b, chromatin was found to be evenly distributed in the nucleus of the cells constituting the control group. The mitochondria appeared full, the ridge structure was normal, and the bilayer membrane structure of the endoplasmic reticulum was found to be normal. In the EPA-PC group cells, euchromatin was concentrated, leading to a decrease in electron density in the nucleus and a small amount gathered around the nuclear membrane of the cell. The vacuolation in the cytoplasm was severe, and the endoplasmic reticulum and mitochondria exhibited different degrees of edema. In EPA-PE-treated cells, the mitochondrial structure changed significantly; the ridge structure showed severe edema and the lysosomes were destroyed.

### Effects of EPA-PC and EPA-PE on the expression of PPARγ-related proteins

**Fig. 4:**
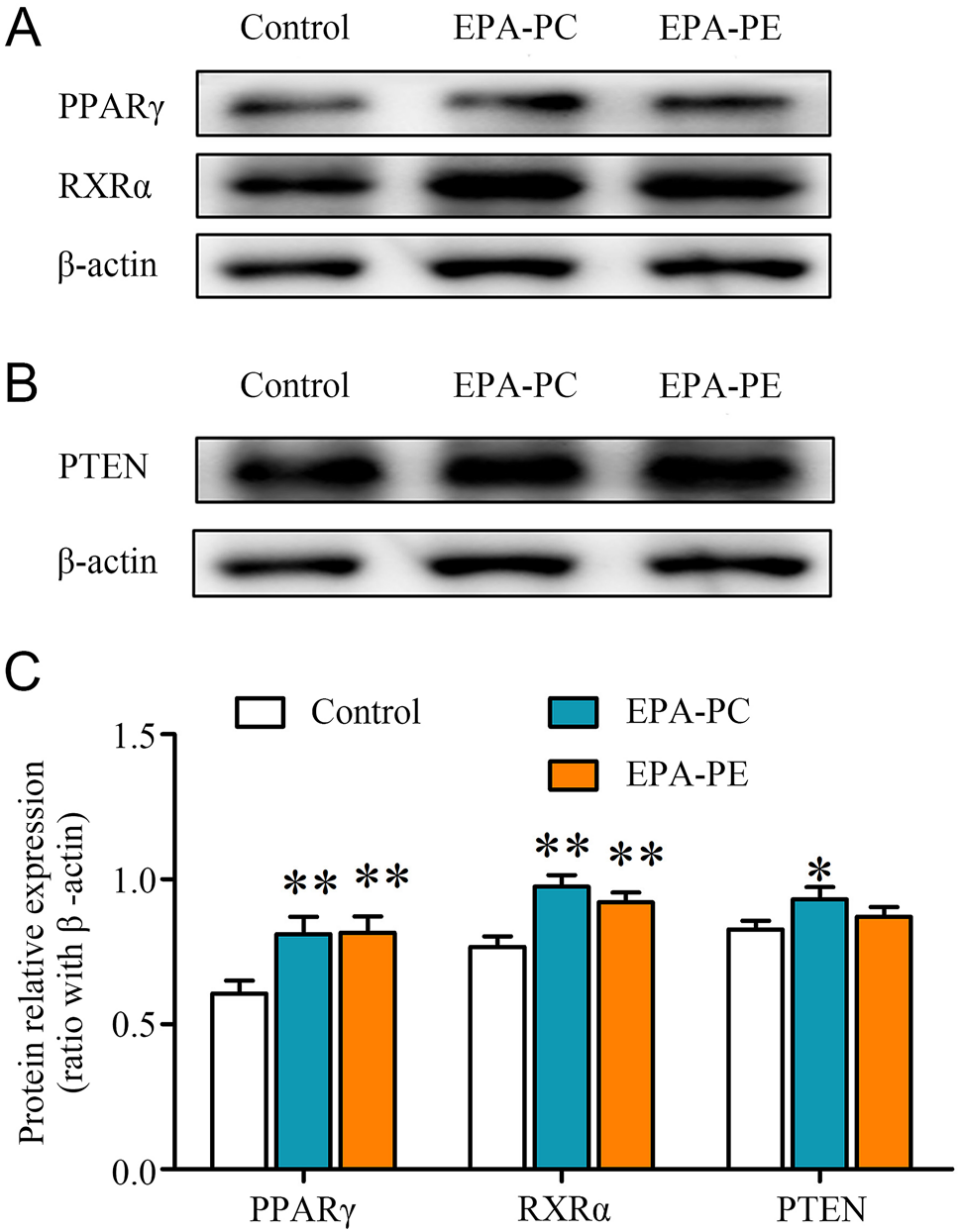
Effects of EPA-PC and EPA-PE on the expression of PPARγ-related proteins in 95D cells. **a** The protein expression of PPARγ and RXRα. **b** The protein expression of PTEN. **c** Quantification of proteins in A, B. β-actin was used as an internal reference. *P < 0.05; **P < 0.01 versus control group. The data are presented as the mean ± SD of three separate experiments

Because EPA is an activator of PPARγ, we determined the protein expression of PPARγ. The results showed that EPA-PC and EPA-PE both upregulated PPARγ expression (Fig. 4a). Retinoid X receptor α (RXRα), which binds to PPARγ to form a heterodimer, was measured using western blot analysis. Compared to the control group, the protein expression of RXRα was significantly upregulated. Phosphatase and tensin homolog deleted on chromosome 10 (PTEN) is the downstream target of PPARγ and also a key tumor suppressor (Teresi et al. 2008). Next, we determined the protein expression of PTEN. We found that the level of PTEN was significantly elevated after treatment with EPA-PC and EPA-PE (Fig. 4b). These results suggested that EPA-PC and EPA-PE might promote the apoptosis of 95D via a PPARγ-related mechanism.

### Effects of EPA-PC and EPA-PE on the expression of the key protein in PI3K/AKT signaling pathway

**Fig. 5:**
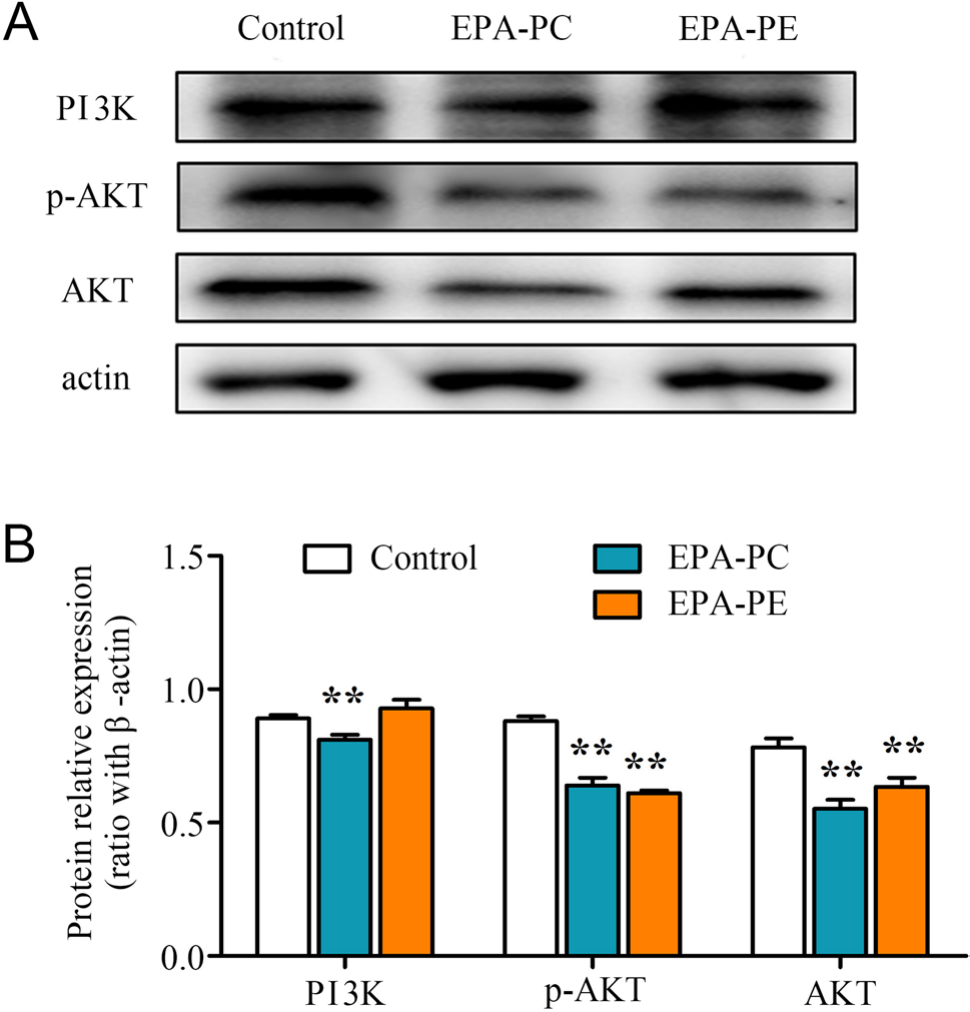
Effects of EPA-PC and EPA-PE on the key protein expression levels of PI3K/AKT signaling pathway in 95D cells. **a** The protein expression of PI3K, p-AKT, and AKT. **b** Quantification of proteins in A. β-actin was used as an internal reference. **P < 0.01 versus control group. The data are presented as the mean ± SD of three separate experiments

PI3K pathway is the target of PTEN and we measured the expression of PI3K/AKT pathway signaling. Results showed that EPA-PC and EPA-PE significantly downregulated the protein expression of PI3K and p-AKT (Fig. 5).

### Effects of EPA-PC and EPA-PE on the mitochondrial apoptotic signaling pathway

**Fig. 6:**
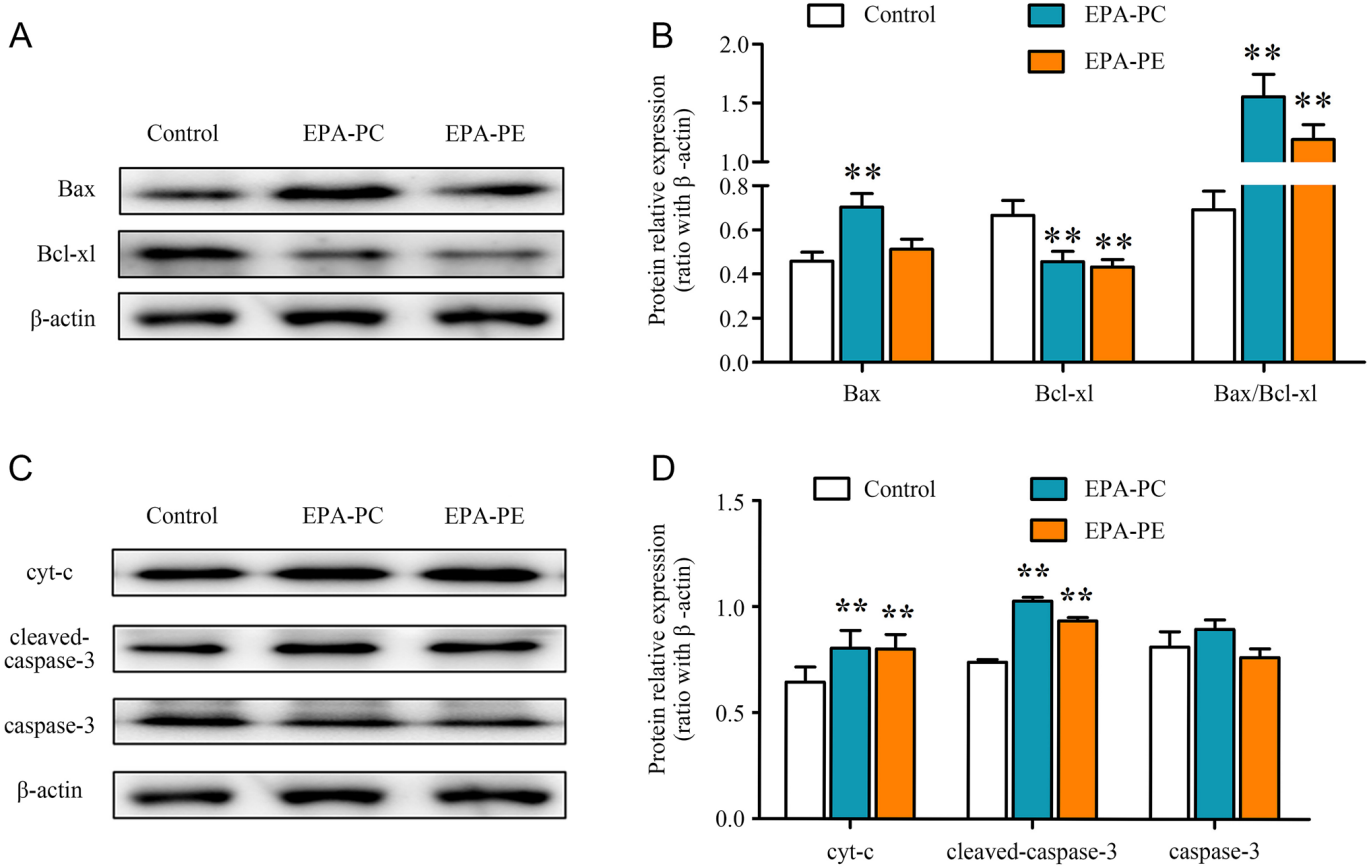
Effects of EPA-PC and EPA-PE on the mitochondrial apoptotic pathway in 95D cells. **a** The protein expression of Bax and Bcl-xl. **b** Quantification of proteins in (**a**). **c** Key protein expression of the mitochondrial apoptotic signaling pathway. **d** Quantification of proteins in (**c**). β-actin was used as an internal reference. **P < 0.01 versus control group. The data are presented as the mean ± SD of three separate experiments

Bax, a pro-apoptotic protein, and Bcl-xl, an anti-apoptotic protein, both belong to the Bcl-2 family. Our results showed an increased Bax expression and decreased Bcl-xl expression after EPA-PC and EPA-PE intervention (Fig. 6a). Responding to damage and stimulation, Bax re-localizes on the mitochondrial membranes and inhibits the function of anti-apoptotic protein, Bcl-xl (Liu et al. 2014). To investigate whether EPA-PC and EPA-PE regulated the mitochondrial apoptotic pathway, we detected the levels of cyt-c in the cytosol. As shown in Fig. 6c, EPA-PC and EPA-PE significantly increased the expression of cyt-c. Furthermore, the level of cleaved caspase-3 expression was elevated. These results suggested that EPA-PC and EPA-PE upregulated the mitochondrial signaling pathway and accelerated apoptosis in 95D cells.

## Discussion

It is generally believed that the omega-3 polyunsaturated fatty acid, EPA, has multiple physiological functions including anti-tumor activity. Marine life is a major source of EPA. The body wall of the sea cucumber is rich in EPA, which is mostly bound to phospholipids. This special form confers several properties upon EPA. In this study, we compared the effects of two different EPA-binding forms, PC and PE, on the proliferation and apoptosis of 95D lung-cancer cells in vitro, and attempted to elucidate their mechanism of action.

In vitro studies have shown that EPA can inhibit proliferation of various cancer cell lines, such as colon, breast, and human cervical carcinoma HeLa cells (Du et al. 2015; Hossain et al. 2009; Zheng et al. 2017). In our study, EPA-PC significantly inhibited the proliferation of 95D cells; however, EPA-PE did not exhibit this effect. Apoptosis is an active and orderly self-destruction process regulated by genes under certain physiological and pathological conditions (Zhang et al. 2019). When apoptosis occurs, the cell morphology and intracellular structure change significantly. Shahid et al. (2017) reported that apoptosis is accompanied by a condensation of the nuclear chromatin, expansion of endoplasmic reticulum, vacuolization, and mitochondrial swelling. Similarly, our results showed that EPA-PC and EPA-PE induced significant structural changes in organelles, such as the ER and mitochondria, which manifested as lumen swelling and internal-folding disorders in 95D cells, indicating that EPA-PC and EPA-PE could promote apoptosis in these cells.

PPARγ is a ligand-dependent nuclear transcription factor (Mueller et al. 2000). When the ligand is activated, a heterodimer of PPARγ and RXRα is formed and modulates target gene expression at the transcriptional level to participate in the regulation of a series of metabolic pathways and physiological activities (Issemann et al. 1990). EPA and its derivatives, as natural PPARγ ligands, elicit good anticancer effects. Rovito et al. (2015) proved that an omega-3 EPA-dopamine conjugate could induce breast cancer cell death through autophagy and apoptosis in a PPARγ-dependent manner. Our results showed that EPA-PC and EPA-PE promoted the expression of PPARγ and RXRα, indicating that PPARγ might play an important role in the inhibition of cancer cell development by EPA-phospholipid.

*PTEN* is a tumor suppressor gene that plays critical roles in cell migration and apoptosis (Lee et al. 2007). It has been reported that multiple PPARγ agonists can upregulate *PTEN* expression in many types of cancer cells including human osteosarcoma cells, hepatoma carcinoma cells, and lung adenocarcinoma cells (Ni et al. 2017; Zhang et al. 2006). PTEN protein regulates phospholipase activity and can dephosphorylate phosphatidylinositol-3,4,5-triphosphate (PIP3), decrease the phosphorylation level of AKT, and negatively regulate the PI3K/AKT signal transduction pathway (Lee et al. 2018). It plays a major role in inducing cell cycle arrest and apoptosis, and regulates cell adhesion, migration, and differentiation (Cai et al. 2018; Yang et al. 2018; Zhu et al. 2017). Cao et al. (2007) found that the PPARγ agonist rosiglitazone could directly increase the expression of PTEN and block the PI3K/AKT transduction pathway to inhibit the growth of HCC cells and induce apoptosis. We obtained similar results and found that EPA-PC and EPA-PE elevated PTEN protein expression by activating PPARγ and repressed PI3K/AKT signaling.

A study by Zhang et al. (2013) reported apoptosis-related protein expression can be regulated by the PI3K/AKT pathway. These authors proved that baicalein could induce apoptosis in EC109 cells by downregulating the anti-apoptotic component, Bcl-2, and upregulating the pro-apoptotic component, Bax, by decreasing the PI3K/AKT signals. Results from our study were similar and indicated that EPA-PC and EPA-PE promoted the expression of Bax and inhibited Bcl-xl. Bax is a sensor for cell damage and stimulation. When apoptosis occurs, Bax inserts into the mitochondrial outer membrane and increases its permeability to induce mitochondrial apoptosis, while anti-apoptotic proteins like Bcl-2 and Bcl-xl play a role in preventing the formation of mitochondrial pores (Zhang et al. 2017; Zhou et al. 2018a, b). A key step of the mitochondrial apoptotic pathway is the release of pro-apoptotic proteins from mitochondria, such as cyt-c (AlBasher et al. 2019). Cyt-c is a protein that transmits electrons in the electron transport chain in the mitochondria. When released into the cytosol, cyt-c binds to apoptotic protease activating factor 1 (Apaf1) to initiate the activation of caspases (Liu et al. 2016). Caspase-3 is a key execution enzyme downstream of the apoptotic cascade and signaling pathway (Maher et al. 2019). Our study showed that EPA-PC and EPA-PE could promote cyt-c release from mitochondria and increase activated caspase-3 protein expression in 95D cells, indicating that targeting the mitochondrial pathway was one of the mechanisms of apoptosis induced by EPA-phospholipid.

In this study, we demonstrated that EPA-PC significantly inhibited proliferation and promoted apoptosis in 95D lung-cancer cells. Further study revealed that the effects involved the PPARγ-dependent PTEN/PI3K/AKT signaling pathways and the mitochondrial apoptotic pathway. EPA-PE had no significant effect on cell proliferation and only slightly promoted apoptosis in 95D cells. The difference in effects between EPA-PC and EPA-PE may be due to the different types of phospholipids to which they are attached. The specific mechanism still needs to be further elucidated.

## Materials and methods

### Extraction of EPA-phospholipids

EPA-phospholipids were extracted from sea cucumber (Zhoushan Fisheries Co., Ltd, Zhoushan, China). The extraction was performed as described by Liu et al. (2017). The fatty acid composition was determined using an Agilent 7820 Gas Chromatograph with a flame-ionization detector. EPA-PC contained 33.20% EPA and 10.50% DHA. EPA-PE contained 28.62% EPA and 3.56% DHA.

### Liposome preparation

All samples used in this study were administered liposomes, which were prepared according to the modified method of Hossain et al. (2006). Equal molar EPA-PC/EPA-PE and cholesterol were dissolved in eggplant bottles with a small amount of chloroform. Subsequently, the mixed system was evaporated into a uniform film by nitrogen blowing, and then, d-Hanks solution was added. After ultrasonic treatment, a milky water suspension formed. Liposomes were obtained by filtration with a 0.22 μm microporous membrane.

### Cell culture

95D cells were purchased from Shanghai Institutes for Biological Sciences (Shanghai, China) and cultured in RPMI 1640 medium (Biological Industries, Kibbutz Beit-Haemeck, Israel) with 10% (v/v) fetal bovine serum (FBS; Biological Industries, Kibbutz Beit-Haemeck, Israel), at 37 ℃ in an incubator containing 5% CO_2_.

### Cell viability assay

95D cells (2 × 10^3^ cells per well) were seeded into 96-well plates and incubated overnight. Then, the cells of treatment groups were treated with medium-containing EPA-PC (5, 25, 50 and 100 μg/ml) or EPA-PE (50, 100, 200, and 400 μg/ml), and the control group was cultured with normal medium without EPA-PC or EPA-PE. After incubation for 24 h, 36 h, and 48 h, respectively, the cells were incubated with thiazolyl blue tetrazolium bromide (MTT, sigma, St. Louis, MO, USA) solution (0.5 mg/ml) for 4 h. Acidic isopropanol was added to fully dissolve formazan, and the absorbance of the solution was measured at the wavelength of 570 nm. The survival curve was plotted and the concentration required to inhibit cell growth by 50% (IC_50_) was calculated (Han et al. 2018).

### LDH activity determination

The LDH content was determined using an LDH kit (Nanjing Jiancheng Bioengineering Institute, Nanjing, China) according to the manufacturer’s instructions.

### Hematoxylin staining

95D cells (3 × 10^3^ cells per well) were seeded into 24-well plates. The cells of treatment groups were incubated with EPA-PC (25, 50 and 100 μg/ml) or EPA-PE (25, 50 and 100 μg/ml) for 24 h, while the cells of the control group were incubated in the medium. Cells were then fixed with 95% alcohol for 20 min and stained with 0.5% (w/v) hematoxylin staining solution (Sigma, St. Louis, MO, USA) for 5 min. After washing thoroughly with running water, diluted hydrochloric acid was added for color separation. Subsequently, the cells were immersed in ammonia water for approximately 5 min until the cells turned blue. Ultimately, the stained cells were placed under a microscope (IX51, Olympus, Tokyo, Japan) for observation and photographing.

### Acridine orange/ethidium bromide (AO/EB) double staining assay

95D cells (3 × 10^3^ cells per well) were seeded into 24-well plates and incubated overnight. The cells of the treatment group were treated with EPA-PC (50 and 100 μg/ml) or EPA-PE (50 and 100 μg/ml) for 24 h, respectively. The cells of the control group were treated with normal medium. The cells were stained with 10 μl/ml of AO (100 μg/ml) and 10 μl/ml of EB (100 μg/ml) for 30 s. The unbound dye was washed with PBS buffer solution. The morphology of cells was identified under fluorescent microscopy (IX51, Olympus, Tokyo, Japan) (Ramalingam et al. 2017).

### Transmission electron microscopy

95D cells were obtained after treatment with EPA-PC (100 μg/ml) or EPA-PE (100 μg/ml) for 24 h. The cells of the control group were treated with normal medium and then fixed with 2.5% glutaraldehyde and dehydrated with ethanol, and cells were stained with uranyl acetate and lead citrate. The ultra-structure of cells was photographed with a JEM-1200EX transmission electron microscope (TEM) and analyzed (JEM-1200EX, Tokyo, Japan) (Shi et al. 2014).

### Western blot analysis

95D cells (1 × 10^6^ cells per well) were seeded into 6-cell plates. The cell treatment group was treated with EPA-PC (100 μg/ml) or EPA-PE (100 μg/ml), while the control group was treated with normal medium. After incubation for 24 h, the cells were lysed with RIPA buffer and the cell lysates were centrifuged at 5000 rpm for 10 min (centrifugal speed for obtaining protein used to detect cytochrome c (cyt-c) was 13,000 rpm). The protein concentration was determined using a BCA kit (Solarbio, Beijing, China) and adjusted to be consistent. The protein samples were separated by sodium dodecyl sulfate-polyacrylamide gel electrophoresis (SDS-PAGE) according to molecular weight differences, and then, the target protein was transferred from the gel to a polyvinylidene fluoride (PVDF; Millipore, Bedford, MA) membrane. Subsequently, the PVDF membrane was blocked with 5% bovine serum albumin at room temperature for 2.5 h and incubated with appropriate primary antibodies including PPARγ, PTEN, PI3K, p-AKT, AKT, Bax, Bcl-xl, cyt-c, and caspase-3 (Cell Signaling Technologies, Danvers, MA, USA) at 4 °C overnight. After washing with 1 × TBST, the membrane was incubated with HRP-conjugated secondary antibody (Proteintech, Rosemont, IL, USA) at room temperature for 2 h. Eventually, the immune blots were visualized by an automatic chemiluminescence apparatus (Tanon 5200, Shanghai, China) and quantified by the ImageJ software (NIH, Bethesda, MD, USA) (Mao et al. 2019).

### Statistical analysis

The results were statistically analyzed by SPSS version 22.0. One-way analysis of variance (ANOVA) was used to detect the differences among the three groups, and LSD pairwise comparison was carried out, with *P* < 0.05 as the statistical difference.
